# Changes in Carabidae Biodiversity in the Perennial Flowering Strip and Adjacent Arable Crops in Organic Conditions

**DOI:** 10.3390/insects17090916

**Published:** 2026-09-01

**Authors:** Jolanta Kowalska, Małgorzata Antkowiak, Paweł Sienkiewicz, Mikołaj Błędkowski

**Affiliations:** 1 Department of Organic Agriculture and Environmental Protection, Institute of Plant Protection-National Research Institute, Węgorka 20, 60-318 Poznan, Poland; m.bledkowski@iorpib.poznan.pl; 2 Department of Entomology and Environmental Protection, Poznan University of Life Sciences, Dabrowskiego 159, 60-594 Poznan, Poland; pawel.sienkiewicz@up.poznan.pl

**Keywords:** agricultural landscape, Carabidae, Coleoptera, population changes, semi-natural habitats, biodiversity indexes, organic crops

## Abstract

Landscape simplification resulting from agricultural intensification threatens biodiversity and ecosystem services on arable land. Semi-natural habitats, such as flower strips, can mitigate these effects by providing habitat and resource diversity and by modifying the microclimate at the agricultural landscape scale. As effective natural enemies in arable fields, ground beetles (Carabidae) play a key role not only in pest regulation, but also in weed control through seed consumption. The study found that FS serve as crucial habitats supporting Carabidae—characterized by a higher Shannon–Wiener index—and foster the presence of rare species absent from adjacent wheat and rapeseed monocultures. Unlike field crops, flower strips exhibit greater evenness in ground beetle community structure, indicating higher ecological stability and superior environmental quality. The research confirmed significant seasonal fluctuations in Carabidae abundance, particularly pronounced in flower strips; an increase in numbers was recorded during May and June in both years of observation, confirming that FS act as stable habitats for these insects.

## Simple Summary

The aim of the study was to evaluate the importance of perennial flower strips (FS) as habitats for Carabidae in an agricultural landscape. The research was conducted in 2024–2025, comparing flower strips with adjacent wheat and rapeseed crops using Barber traps. Flower strips exhibited higher diversity (Shannon–Wiener index (H’) for FS was 2.108 and 2.481, respectively, in 2024 and 2025), the presence of rare species, and greater assemblage evenness compared to monocultures (H’ for winter wheat was recorded as 1.724 and 1.596 in 2024 and 2025; for rapeseed was, respectively, 1.701 and 1.233). An increase in Carabidae abundance was also observed over the course of the study months. The results indicate that FS can serve as stable habitats supporting biodiversity in agroecosystems, particularly amidst intensifying climatic anomalies.

## 1. Introduction

Intensive agriculture, characterized by the combination of monocultures and synthetic inputs, negatively affects insect diversity and abundance. Agricultural intensification leads to landscape simplification and a decline in biodiversity, particularly among invertebrates that perform key ecosystem functions. Ground beetles (Carabidae) inhabit most terrestrial ecosystems and play crucial roles; notably, many species are important predators that contribute to natural pest control in agricultural landscapes [1]. Carabids are recognized as important bioindicators of environmental changes and agricultural habitat quality, as they respond to both vegetation structure and microclimatic conditions [2]. To mitigate the negative impacts of monocultures, measures that enhance environmental heterogeneity—such as perennial flower strips—are increasingly being implemented; these strips can serve as refugia and food sources for numerous organism groups. Such semi-natural habitats can provide diversity, resources, and landscape-scale climate modification, while also influencing the population dynamics of epigeic organisms (those living on the soil surface)—including Carabidae—especially in the context of changing climatic conditions [3]. Flower strips (FS) are increasingly being adopted within agri-environmental schemes, highlighting their immense potential and multifunctionality [4].

Studies highlight the crucial role of flower strips in enhancing arthropod diversity within agricultural landscapes. Flower strips adjacent to crop fields are positively associated with the density of spiders and ground beetles (Carabidae), while vegetation characteristics within the strips significantly influence arthropod community traits [5]. As effective natural enemies in arable lands, ground beetles play a key role in regulating not only pests but also weed seeds. Field management practices influence their communities, while landscape factors affect them both directly and indirectly—for instance, through air temperature [6]. Recent research on agricultural intensification [7] indicates that the dominance of generalist species in monocultures reflects the process of biotic homogenization—an increase in the similarity of species composition across distinct, often widely separated ecosystems—leading to large-scale faunal and floral uniformity.

Research on the role of flower strips in shaping Carabidae communities can provide important information, both from a theoretical perspective and with practical implications. However, current data on the functioning of perennial flower strips in Central European conditions are still lacking. From a practical perspective, such results may have direct implications for the design of sustainable agricultural production systems, and increasing their diversity and population stability may translate into reduced pesticide use. Therefore, such research can provide a basis for developing effective agricultural landscape management strategies, supporting both biodiversity conservation and the stability of agricultural production.

The aim of this study was to assess the role of flower strips as semi-natural habitats in shaping the diversity, structure, and seasonal dynamics of Carabidae communities on arable land, as well as to determine their significance in mitigating the negative effects of agricultural intensification on biodiversity, particularly in comparison to adjacent crop monocultures (rapeseed and winter wheat).

## 2. Materials and Methods

### 2.1. Study Area

The study area consisted of a 6 m wide and 100 m long flowering strip (FS) established in the spring of 2021 between a field of winter rapeseed (*Brassica napus* L. var. *napus*) and a field of winter wheat (*Triticum aestivum*) in rotation, where no chemical pesticides or synthetic nitrogen fertilizers were used, at the Winna Góra Experimental Station (52.209963, 17.433402) in western Poland (Figure 1).

It was sown with a commercial mixture Bees’ universe Toraf (commercially available on the market) containing seeds of 30 species of undemanding annual, biennial and perennial plants with high nectar and pollen yield (Figure 2). The seeds were sown to a depth of 1 cm and then rolled. In each year, in autumn, 50% of flower strip area was mowed. The strip and fields are located on moderately good arable soils (soil quality class IIIb), predominantly belonging to the good rye agricultural suitability complex (complex 5). The FS has not been mowed or threatened with pesticides.

The study, as described in this manuscript, involved an assessment of the flower strip in the fourth and fifth years following its establishment, as well as an analysis of the Carabidae population in the flower strip and the adjacent crops, which are in line with the crop rotation practiced and have not previously been analyzed or described. The study site described is completely new from an agricultural, botanical and arthropod biodiversity perspective compared to another paper of our research team.

### 2.2. Survey of Carabidae

An inventory of carabids in the FS and adjacent organic fields was made. It provided a preliminary picture of their diversity both in the FS and in the adjacent fields, i.e., in the rapeseed and winter wheat fields. Observations were conducted for two growing seasons (2024 and 2025), on a multi-year flowering strip (in the fourth and fifth year after establishment). Beetles were collected eight times in each season: four times in May and four times in June, every one week in each month. The material was collected in ground traps filled with a preservative fluid (70% ethylene glycol with a drop of liquid detergent to minimize surface tension) in five samples per each date and location. The Barber traps with volume 500 mL were placed in the center of the flower strip, along its entire length, maintaining a constant distance between the traps as 20 m., a total of 5 traps were used for each variant/habitat. Distance between traps and number of traps per site are fitted to field circumstance and to omit problems with pseudoreplication and overfishing of ground beetles. Same scheme and number traps were placed similarly in the fields immediately adjacent to the strip (Figure 3).

### 2.3. Location and Weather Conditions

To determine the environmental variables important for the abundance of Carabidae populations, meteorological data from the Winna Góra Experimental Station were used in the period from January 2024 to June 2025. The crops were in rotation; this means that in 2025 the rapeseed and wheat fields were swapped compared to 2024. In both growing seasons, these crops were adjacent to same flower strip. Average air temperature in Poland in 2024 was 10.9 °C, which was 2.2 degrees higher than the long-term annual average (based on the 1991–2020 climatological normal period). Key climate characteristics of Winna Góra in 1991–2020 (regional weather data) indicate that the warmest month was July, with an average daily temperature of 19.5 °C, the coldest month was January, with an average of −0.4 °C. Annual average was approximately 9.9 °C, which places this region among the warmest areas in Poland. According to the Institute of Meteorology and Water Management—National Research Institute (IMGW), 2024 is classified as an extremely warm year based on the average value for Poland. It was also the warmest year in the history of instrumental measurements in Poland. The average air temperature in 2025 was slightly lower, at 9.5 °C, although, like 2024, it is classified as a very warm year [http://www.imgw.pl/, access on accessed on 17 August 2026]. As for the average air temperature in the study area for the months when traps were collected—May and June—the temperatures were 17.1 °C and 18.6 °C in 2024, and 11.6 °C and 17.8 °C in 2025. The average total precipitation in Poland in 2024 was 607.8 mm, representing nearly 99.4% of the norm established from 1991 to 2020 measurements, thus classifying 2024 as an average year. In 2025, this value was lower—namely 546 mm—representing 89.31% of the long-term average, which classifies the year as dry [http://www.imgw.pl/, accessed on 17 August 2026] (Figure 4).

### 2.4. Statistical Analysis

The results were statistically processed using the Statistica 12.0 program (StatSoft Polska, Kraków, Poland). Prior to the analysis, the Shapiro–Wilk test for normal distribution was performed. Data did not have a normal distribution, the Kruskal–Walli’s test (a non-parametric equivalent to one-way ANOVA) or U Mann–Whitney test (nonparametric equivalent of Student’s *t*-test) was performed. Multiple comparison tests were performed. The number of beetles in each trap, in each field/habitat and each date was analyzed. An analysis of species diversity between the flower strip and rapeseed and wheat fields was conducted using the Shannon–Wiener Index, while Pielou’s Evenness Index was calculated to quantitatively assess and compare the dominance structure in Carabidae communities across the habitats and sampling dates, calculated with the program PAST 4.03 [8].

## 3. Results

### Survey of Carabidae

Analysis of Carabidae abundance revealed distinct seasonal population dynamics and varied habitat preferences. In 2024, the most abundant species across all habitats was *Poecilus cupreus*; its share of the population peaked in June—particularly in the flower strip, where 216 individuals were recorded that month. In June, *Amara tricuspidata* was also highly abundant in the wheat field (342 individuals). Overall, carabid abundance increased over time at every site, reflecting the seasonal activity and population growth of ground beetles (numbers were consistently higher in June than in May). While similar (average) numbers of individuals were recorded in the flower strip and the wheat field, the flower strip exhibited greater species diversity; indeed, certain species were observed exclusively there—such as *Amara aulica*, *Amara familiaris*, *Anisodactylus binotatus*, *Dromius linearis*, *Harpalus froelichii*, *Harpalus griseus*, *Nebria brevicollis*, and *Agonum emarginatum* (though the latter is a species associated with riparian habitats, and its presence in the FS was likely accidental). The population was dominated by a few species, while others appeared only sporadically. Some of these are considered relatively rare or atypical for intensive agroecosystems—being less frequently encountered in cultivated fields—which makes them ecologically significant, and examples include *Dromius linearis* and *Agonum emarginatum* (Table 1).

Similar patterns were observed in the following year of the study (2025). The analysis confirmed distinct differences in Carabidae community structure among the study sites (habitats); crop fields (especially rapeseed) were characterized by very high individual abundance alongside strong dominance by a few species—primarily *Poecilus cupreus*, which in some areas accounted for over 60–80% of the total. The flower strip exhibited lower dominance by individual species and a more even distribution of the various taxa. At the same time, the highest number of species (35) was recorded in the flower strip, including species rarely found in intensively managed agroecosystems, confirming its role as a biodiversity refuge. As in the previous year (Table 1), several species were observed exclusively in the flower strip—namely *Amara familiaris*, *Amara spreta*, *Calathus melanocephalus*, *Harpalus froelichii*, *Pterostichus oblongopunctatus*, *Stomis pumicatus*, and *Syntomus truncatellus*—with the last two being particularly associated with less modified habitats, thereby indicating the high environmental quality of the flower strip (Table 2).

A comparison of ground beetle (Carabidae) species richness between the flower strip and adjacent crops revealed that, in 2024, the highest Shannon–Wiener index was recorded in the FS in May (2.196), while the lowest was in the rapeseed field, in May (1.344). The index for rapeseed fields in June compared to May was increased to 2.057. In June, species diversity in the FS remained at a similar level (2.019). In the wheat field in May the Shannon–Wiener index was 2.004 and in June 1.444. When considering all data collected over both months, the highest diversity was recorded in the FS (mean 2.108), followed by the wheat field (mean 1.724) and the rapeseed field (mean 1.701). A similar trend over both months was observed in 2025, with even more pronounced differences (mean for FS: 2.481; wheat: 1.596; rapeseed: 1.233). In the subsequent season of use, the flower strip exhibited higher diversity, whereas the adjacent fields showed significantly lower diversity. 

An analysis of Pielou’s evenness index in 2024 revealed varying dynamics for the index (*J’*) depending on the habitat. The flower strip exhibited the highest structural stability, with index values remaining virtually unchanged (*J’* = 0.652 in May and *J’* = 0.627 in June); this confirms its role in limiting the dominance of individual taxa and supporting balanced, multi-species assemblages. A different trend was observed in the adjacent monoculture fields. In the wheat field, a sharp decline in evenness was noted—dropping from 0.740 in May to 0.500 in June—clearly indicating progressive monopolization by dominant species as the crop developed. Conversely, the rapeseed field showed the opposite trend—an increase from 0.646 to 0.699, suggesting a reduction in competitive pressure from the dominant species following the crop’s peak flowering phase. Significant changes in the Carabidae community structure were recorded in 2025 compared to the previous year. The most pronounced differences occurred in the rapeseed field, where Pielou’s evenness index dropped drastically (to 0.335 in May and 0.455 in June). A shift in trends was also observed in the flower strip (a decline in evenness from 0.855 in May to 0.715 in June). In the case of winter wheat, however, last year’s trend was reversed: the indicator remained at a stable, moderate level, with a slight increase in June (Figure 5).

An analysis of number species in 2024, regardless of the month, reveals a statistical proofed advantage within flower strips (mean number 5.13) compared to winter wheat (mean number 2.35) and rapeseed fields (mean number 2.05). In 2025, the FS exhibited the highest mean number species (5.13); however, this average was statistically comparable to that of the rapeseed field (Figure 6). 

Regarding monthly observation, the species richness of the flower strip in 2024 was statistically higher only in May compared to that of rapeseed (1.05). In May 2025, no differences were observed between habitats, whereas June (very hot month) stood out in particular—the average species richness in the FS habitat reached as high as 8.75, which was a statistically significantly higher value compared to the wheat field (2.08) (Figure 6).

Statistical analysis of the obtained data revealed a tendency for Carabidae abundance to vary across habitats—particularly favoring flower strips, though this effect was limited by high data variability. In 2024, a significant effect of habitat on ground beetle abundance was observed (Kruskal–Walli’s test: χ^2^ = 8.94, *p* = 0.0114). The highest mean ranks were recorded in the flower strips, indicating a tendency for assemblages with higher beetle abundance to persist in this habitat compared to wheat or rapeseed fields. However, the absence of significant differences in post hoc analyses suggest that the differences between habitat pairs were not sufficiently pronounced. In 2025, no significant effect of habitat on ground beetle abundance was found (χ^2^ = 1.60, *p* = 0.4493). Despite a high mean value in the rapeseed fields, substantial data variability and a wide range of values indicate strong within-group variation, resulting in a lack of statistically significant differences between habitats (Table 3).

An analysis of the mean abundance of Carabidae in 2024 (regardless of habitat) revealed a significant increase from May to June (Mann–Whitney U test: U = 315.5, Z = −2.03, *p* = 0.043). Ground beetle abundance was significantly higher at the later date, reflecting seasonal population dynamics independent of habitat type. However, when analyzing each habitat separately, no significant difference in abundance between May and June 2024 was observed in the wheat field (Mann–Whitney U test: U = 40.50, Z = −0.73, *p* = 0.467). The observed increase in abundance in June (evident in the mean values) was not pronounced enough to be statistically confirmed (a clearly noticeable upward trend existed, but data variability rendered the difference statistically non-significant). A significant increase in ground beetle abundance from May to June was found only in the flower strip (Mann–Whitney U test: U = 15.00, Z = −2.61, *p* = 0.467). No statistically significant differences were found in the rapeseed crop between May and June 2024 (Mann–Whitney U test: U = 37.50, Z = −0.90, *p* = 0.332). In 2025, a pooled analysis across all habitats revealed no statistically significant difference in Carabidae abundance between May and June (Mann–Whitney U test: U = 353.5, Z = −1.45, *p* = 0.148), despite a larger difference in the mean values. That season, no significant change in ground beetle abundance was observed in the wheat field (Mann–Whitney U test: U = 49.50, Z = 0.00, *p* = 1.000) or the rapeseed field (Mann–Whitney U test: U = 40.50, Z = −0.73, *p* = 0.463). In contrast, a very marked increase in ground beetle abundance was recorded in the flower strip during this period (Mann–Whitney U test: U = 0.00, Z = −3.74, *p* = 0.000), like the previous year (Table 4).

## 4. Discussion

Analysis of the seasonal dynamics and population structure of Carabidae revealed significant differences in abundance and species diversity between the flower strip and cultivated fields; this aligns with observations by another author highlighting the role of diverse habitats in maintaining biodiversity within agricultural landscapes [9]. As indicated by Penel et al. [10], agricultural environments act as a strong environmental filter, favoring species already adapted to frequent and intense disturbances. These conditions include, among others, regular fertilization and the use of chemical agents, which limit the presence of more sensitive species. While climatic factors appear to be the primary drivers of community composition, species richness seems to depend more on local vegetation characteristics, such as vegetation height and plant species composition. The dominance of *Poecilus cupreus* and *Amara tricuspidata* across various habitats (Table 1) confirms their adaptability, manifested—for instance—through the development of starvation-resistance mechanisms in response to agricultural pressure [6,11,12]. Results from Uroy et al. [6] demonstrated that landscape heterogeneity influences ground beetle activity—specifically density and composition—which in turn affects weed seed predation. In studies by Kowalska et al. [13] Carabid species diversity was higher in field strips than in cultivated fields, and the most abundant epigeic beetle was *Harpalus rufipes* (De Geer), a generalist predator that also feeds on weed seeds. Grasslands, by providing resources and shelter, favor ground beetles, but they also appear to be associated with higher maximum air temperatures, which may influence community composition through thermal tolerance traits [6]. Greater floristic diversity in flower strips and the presence of perennial plants (e.g., *Trifolium pratense*) increase the availability of food resources and microhabitats. Flower strips are characterized by higher Carabidae species diversity than adjacent crops, and vegetation structure (density, cover, flowering phenology) influences the abundance and functional composition of this arthropod group. However, the selection of appropriate species compositions for flower-strip seed mixtures should take into account not only the preferences of beneficial insects but also environmental conditions [14]. More complex vegetation (varying plant heights and types) enhances habitat diversity, benefiting both predatory and omnivorous species [1,5,14]. Specific insect preferences for particular plants are also evident [15].

Higher species diversity and the presence of rare, habitat-specialized taxa—such as *Syntomus* spp., which are confined to flower strips—underscore the value of these enclaves as habitats supporting the conservation of species less resilient to intensive agriculture. Findings by Peterson et al. [16] demonstrated that Carabidae diversity peaked during the rainy season and was lowest at the end of the dry season (in Tanzania); while significant differences existed across the three seasons, the compositional similarity of the assemblages between them was low. The authors conclude that Carabidae assemblages in human-impacted areas fluctuate seasonally, necessitating the alignment of agricultural operations (such as strip mowing) with the insects’ life cycles. Similarly, Grabener et al. [1] indicate that differences in abundance between crop fields (in this instance, maize) and flower strips may be even more pronounced during the autumn months (post-harvest) and winter, provided that flower strip vegetation remains in the field.

The results indicate that FS foster increased ground beetle (Carabidae) diversity, whereas agricultural crops—particularly rapeseed—promote simplified assemblages dominated by a few species; this may have significant consequences for the functioning and stability of agroecosystems (Table 1 and Table 2). The upward trend in species richness from May to June, particularly evident in the FS, demonstrates the habitat’s beneficial impact on maintaining and developing biodiversity throughout the growing season. These findings confirm the significant role of flower strips in enhancing the structural and functional diversity of agricultural ecosystems, which is crucial for sustainable landscape management and the support of natural ecological processes (Figure 6). The higher Carabidae species diversity observed in the flower strips—expressed by the Shannon–Wiener index (mean *H’* of 2.108 in 2024 and 2.481 in 2025)—underscores the pivotal role of these structures as biodiversity refuges within a landscape dominated by rapeseed and wheat monocultures. In both years, the flower strip exhibited not only the highest index values but also relative stability in these values across months, suggesting the maintenance of favorable habitat conditions over a prolonged period of the growing season. Rapeseed and wheat fields exhibited lower values with greater variability, potentially indicating more stressful conditions for these organisms (Figure 5 and Figure 6, Table 3 and Table 4). Of particular note is the increase in the diversity index during the second year of the strip’s existence; this aligns with findings by Blaauw and Isaacs [17], who demonstrated that as wildflower strips age, an accumulation of ecological niches occurs, facilitating the colonization of species with narrower habitat specializations. At the same time, the authors point out that over time, these plantings can contribute to increased yields, provide a return on the initial seed investment, and safeguard crops against pollinator loss. Although the aforementioned studies focus on bees, they demonstrate that the age of a wildflower strip enhances its ecological value. The stark contrast between the wildflower strips and the cultivated fields (where a decline in diversity to 1.233 was recorded in rapeseed in 2025) is consistent with research by Haaland et al. [18], which identifies wildflower strips as essential components for mitigating the negative impact of agricultural intensification on epigeic fauna. The stability of the diversity index within the FS throughout the season (May–June) suggests that they serve as a permanent habitat, unlike cultivated fields, where dynamic changes may be driven not only by agronomic practices but also by the phenological stages of the crop plants.

In the present study, the flower strip was 6 m wide, a dimension determined by a prior review of relevant literature [19,20,21]. A study by Li et al. [5] investigated how the width of flower strips adjacent to maize fields affects the diversity of spiders and ground beetles. Over a two-year period, flower strips of three different widths—2 m, 4 m, and 6 m—were compared with areas dedicated solely to maize cultivation. The results showed that wider FS (4 m and 6 m) supported more diverse and abundant spider and beetle communities compared to narrower strips and areas used exclusively for maize. Spider activity was higher near the strip edges, with the greatest activity recorded in the 4 m wide strips, and vegetation characteristics played a key role in shaping arthropod communities. These findings highlight the importance of designing wider strips to enhance biodiversity and support natural pest control on agricultural land, thereby contributing to the goals of sustainable agriculture.

Not only habitat conditions, but also the pattern of temperature and precipitation during the study years can significantly shape the structure and dynamics of Carabidae assemblages. In our study, the extremely warm year of 2024 and the very warm—albeit drier—year of 2025 (Figure 4) may have favored increased ground beetle activity, particularly during the spring, as reflected in the marked rise in abundance between May and June. At the same time, flower strips—likely due to greater plant diversity (Figure 2) and more stable microclimatic conditions (e.g., higher moisture retention and reduced temperature fluctuations)—may have acted as a buffer against less favorable weather conditions, especially during the dry year of 2025. Consequently, higher species diversity, greater assemblage evenness, and the presence of stenotopic and rare species were observed there, in contrast to structurally simplified monocultures dominated by eurytopic species such as *Poecilus cupreus*. The results indicate that FS can serve as stable refuges for Carabidae, particularly amidst intensifying climatic anomalies.

## 5. Conclusions

1. Flower strips constitute a key habitat supporting Carabidae, characterized by a higher Shannon–Wiener index and the presence of rare species (such as *Stomis pumicatus*) that were absent from adjacent winter wheat and rapeseed monocultures. In contrast to field crops—where a strong dominance of eurytopic species (characterized by a broad ecological tolerance range, e.g., *Poecilus cupreus* accounting for up to 80% of the total) was observed—the flower strip exhibited greater evenness in assemblage structure (with species distributed more proportionally and in similar numbers), indicating higher ecological stability and superior environmental quality within these enclaves.
2. However, the analysis of Pielou’s evenness index revealed variable taxon dominance dynamics that were susceptible to seasonal factors. In both years of observation (2024–2025), an increase in Carabidae abundance was also recorded between May and June, suggesting that flower strips serve as stable habitats during critical developmental stages of these insects.
3. The dominance of generalist species in crops reflects the large-scale homogenization of species composition. Carabids—a group sensitive to environmental changes yet responsive to habitat enrichment (e.g., through wildflower strips)—can counteract this process. The presence of more demanding species in FS enhances the biodiversity of the agricultural landscape.
4. Carabid beetles serve a dual function: They act both as bioindicators of environmental quality and as active agents supporting environmental stability. In landscapes dominated by monocultures, flower strips can help maintain diversity, curbing homogenization and supporting the functioning of agroecosystems in a more sustainable manner.

## Figures and Tables

**Figure 1:**
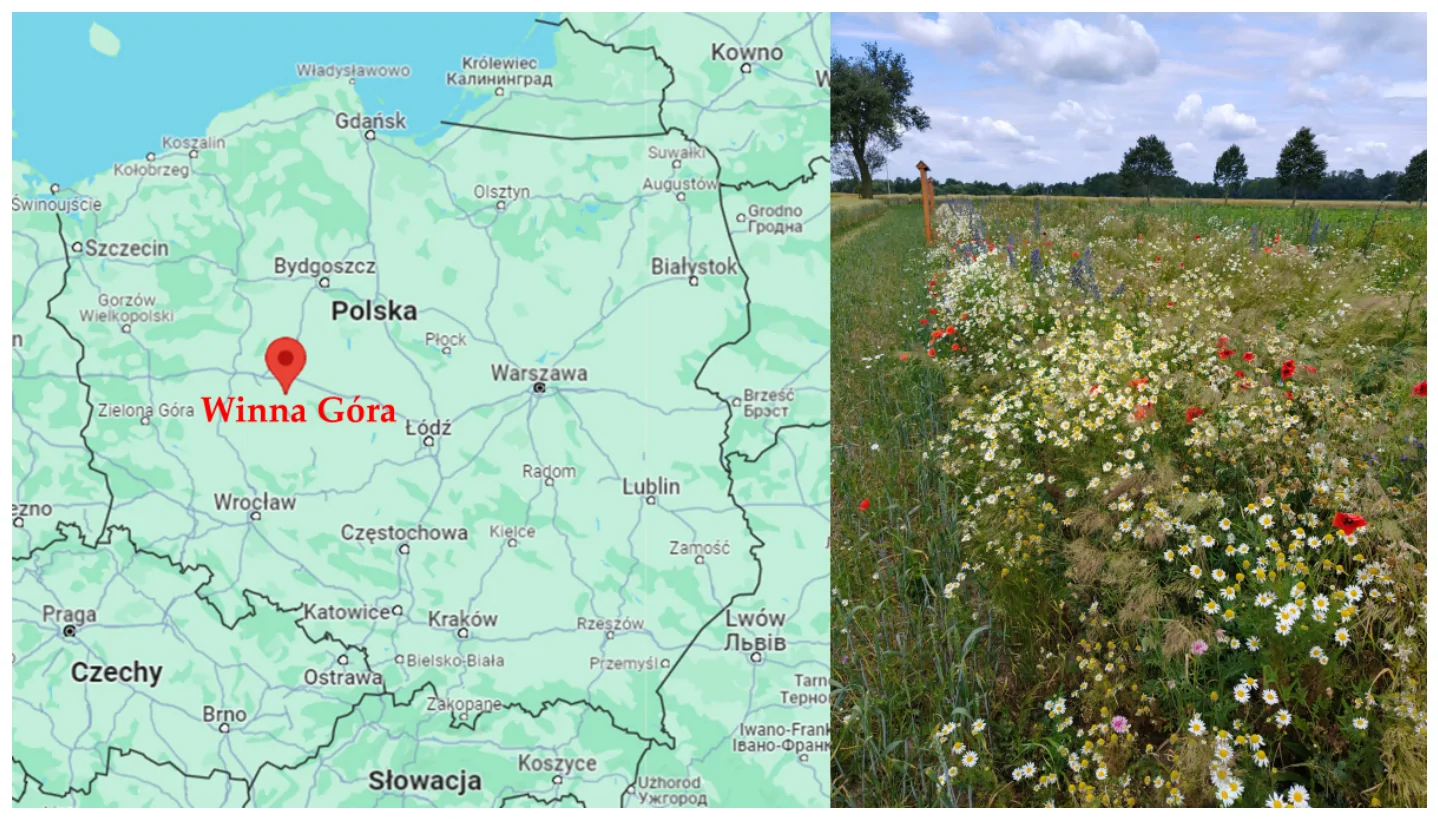
Location of the flower strip and study fields (own edition based on Google map (CC0), 2024–2025, on the right—flower strip.

**Figure 2:**
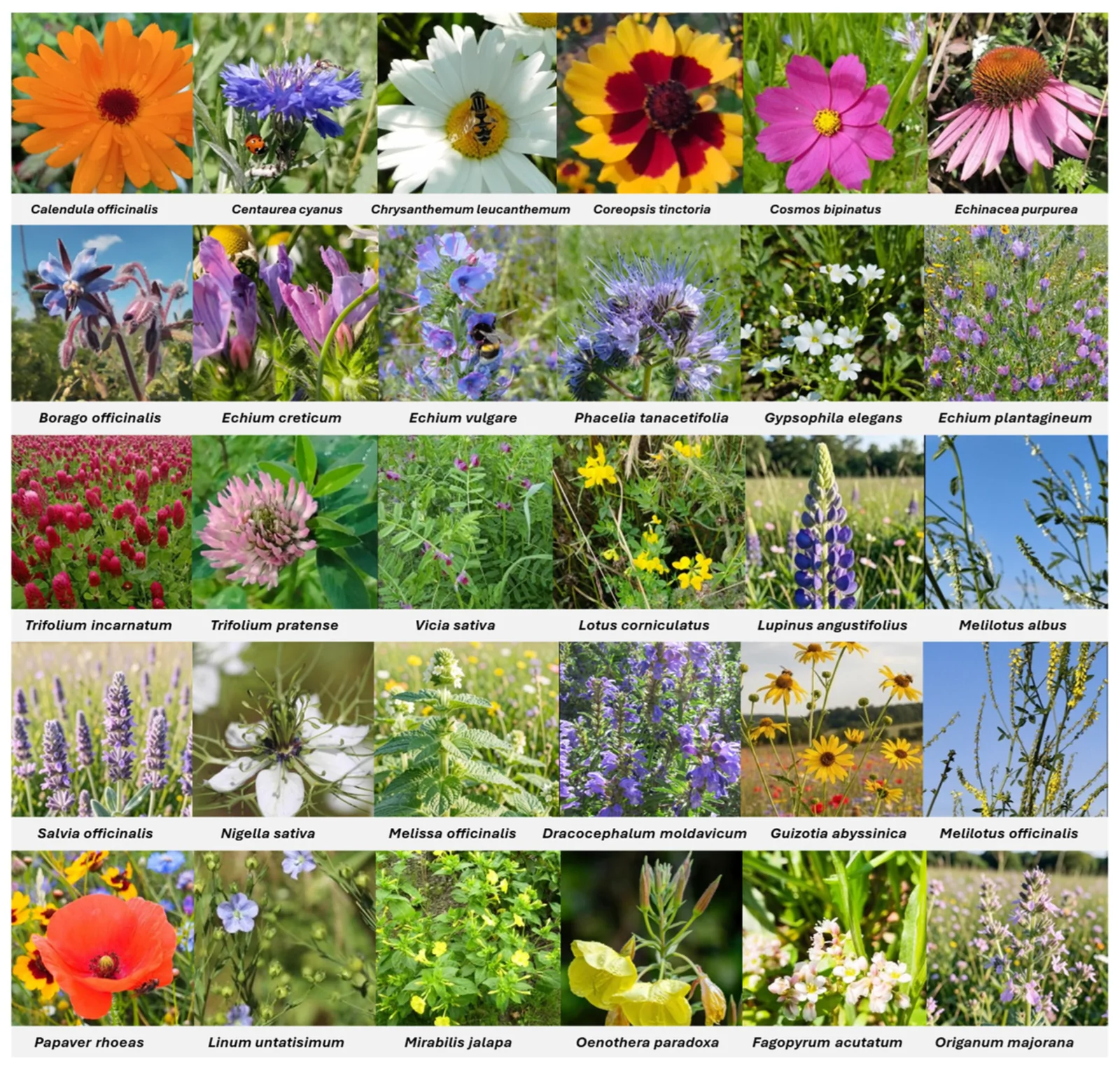
Plants sown as a commercial mixture (2021). Changes in the species composition of plants in the flower strips during the years covered by the study are presented in another article by the same authors (photos: M. Antkowiak).

**Figure 3:**
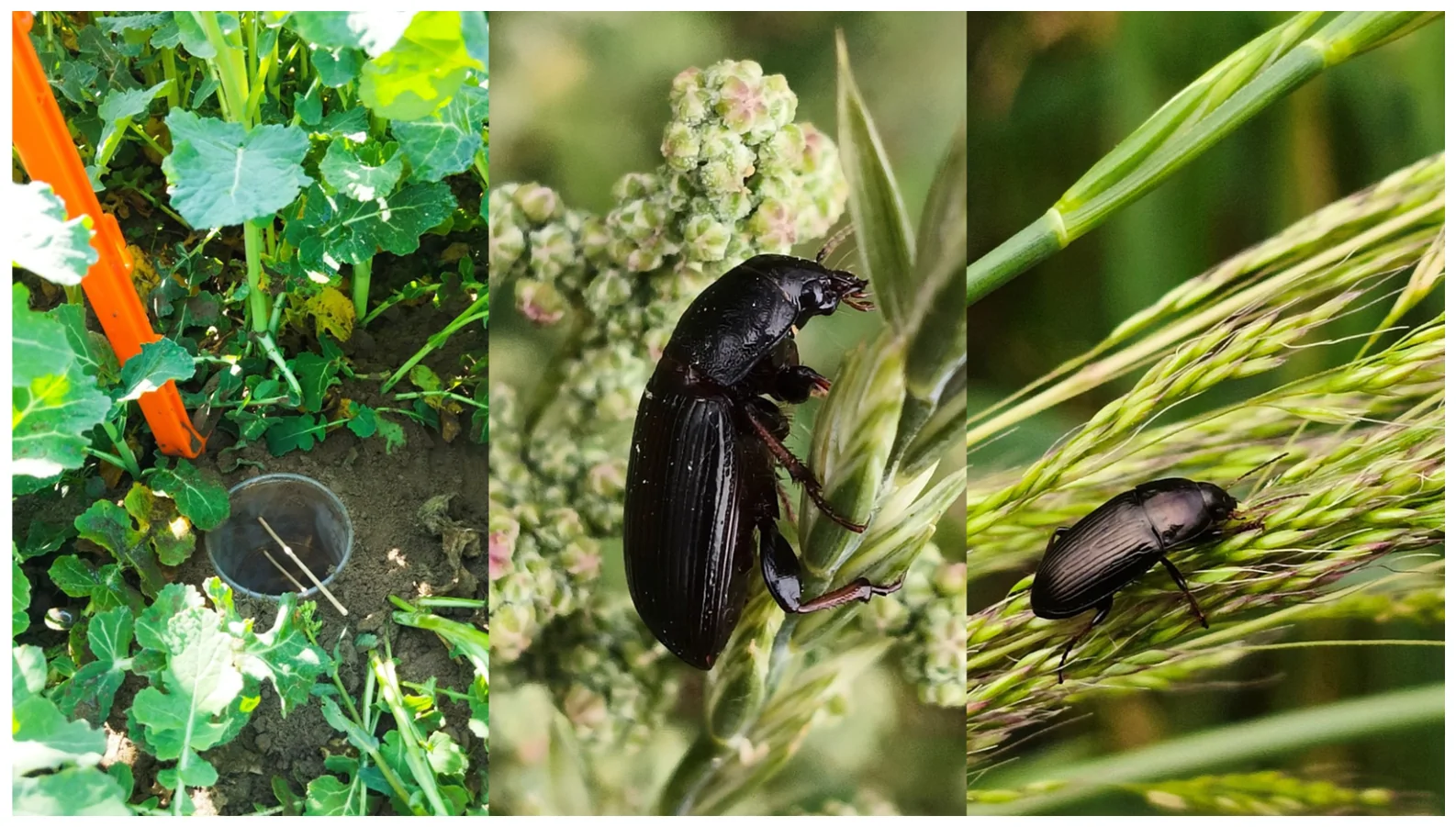
Trap located in a rapeseed field adjacent to the flower strip and examples of individuals—*Zabrus tenebrioides* and *Amara tricuspidate* on the plants (from the left).

**Figure 4:**
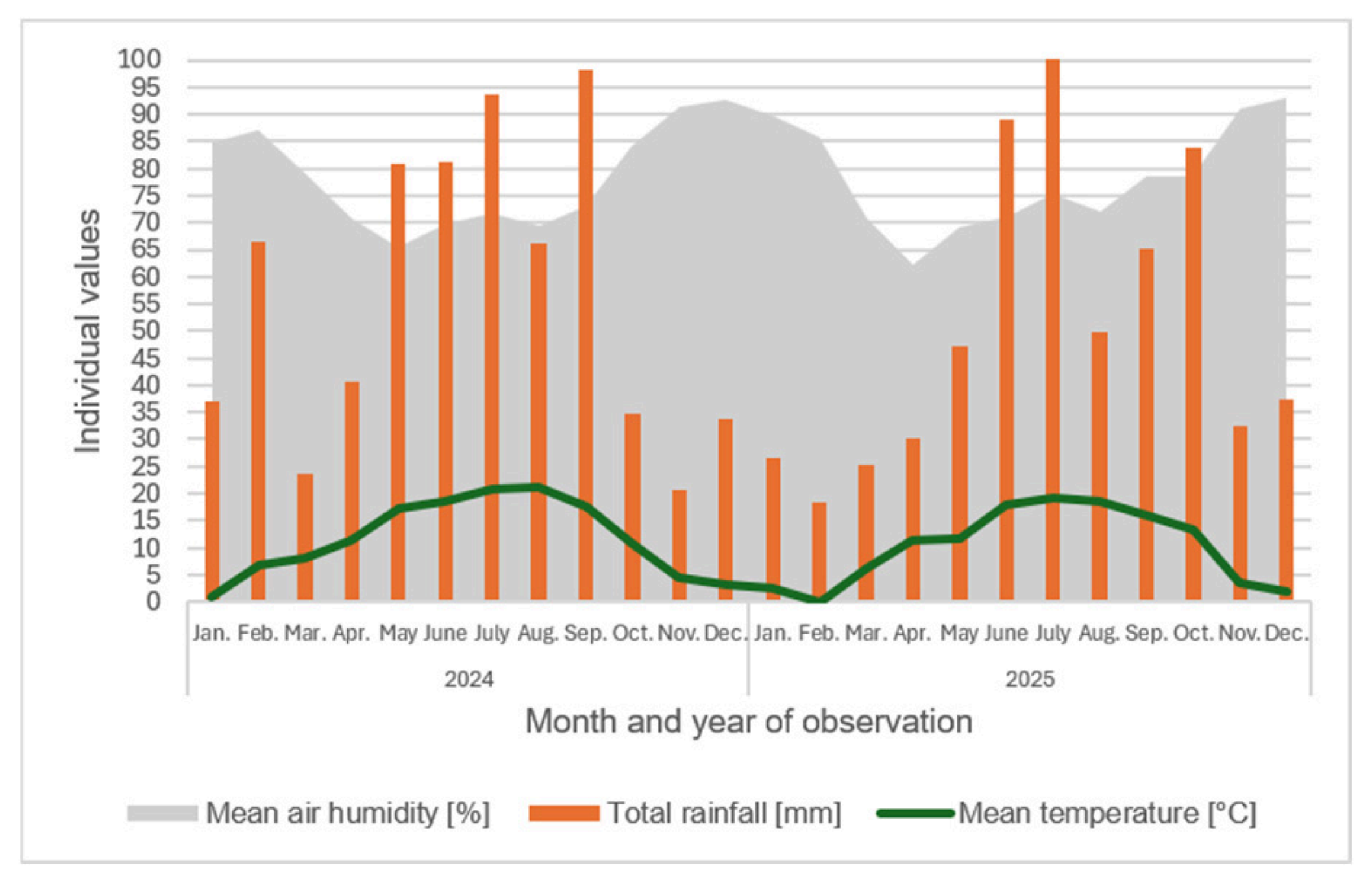
Weather conditions in the fourth (2024) and fifth (2025) year after establishing the flower strip.

**Figure 5:**
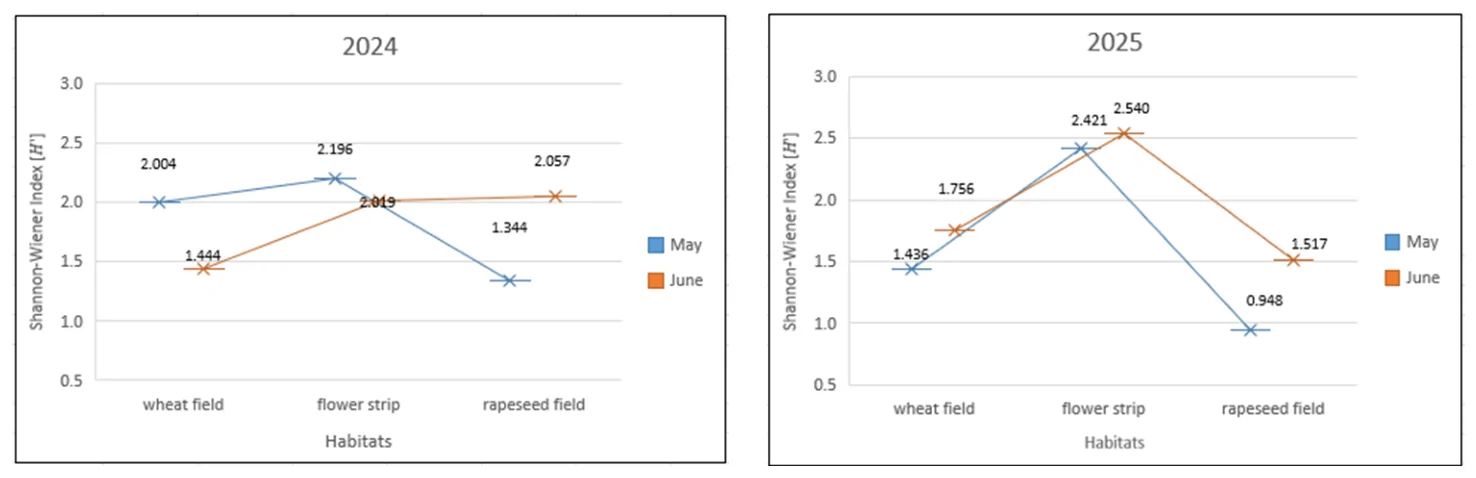

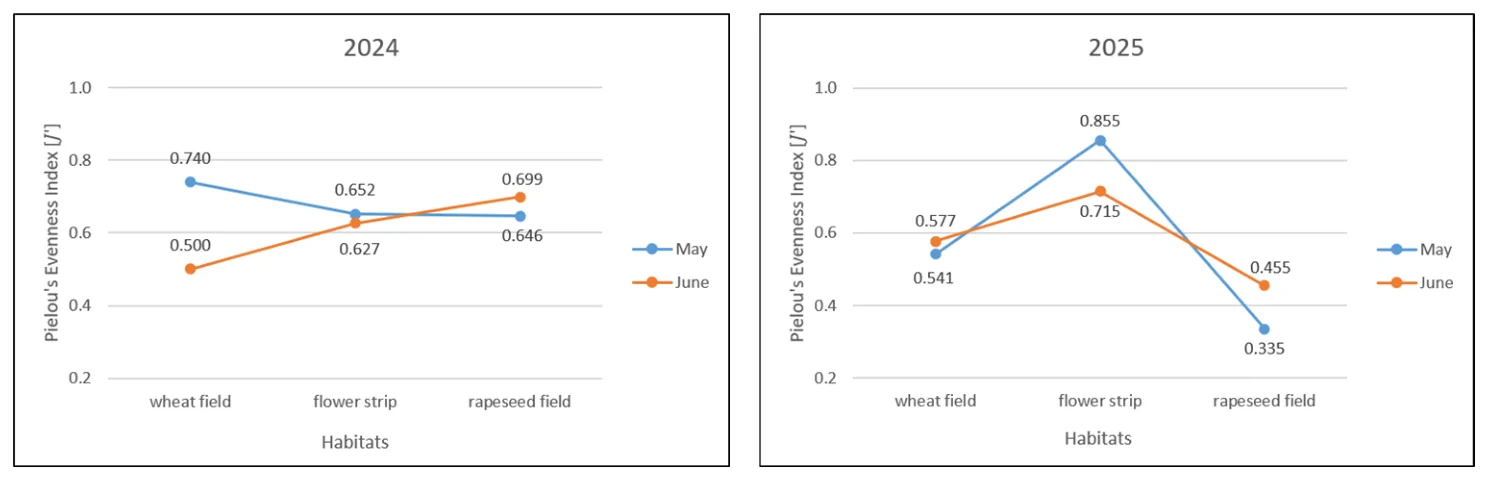
Shannon–Wiener Index (*H’*) and Pielou’s Evenness Index (*J’*), depending on habitats and the date of inventory.

**Figure 6:**
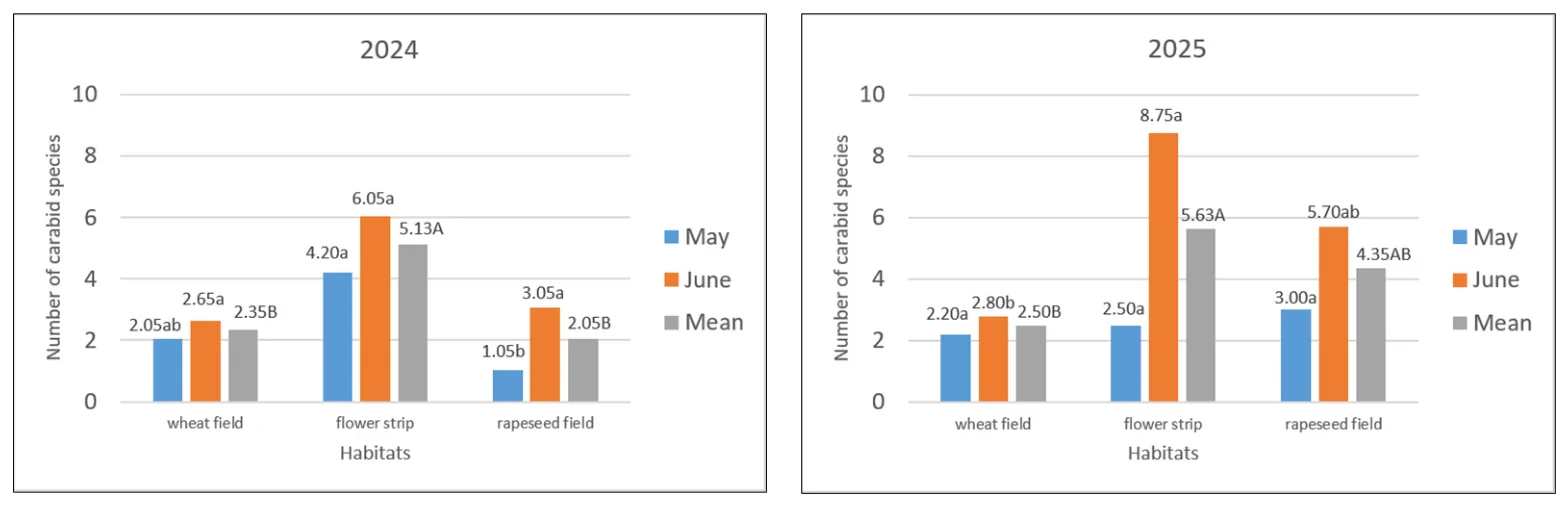
The mean number of carabid species, depending on habitats and month of inventory. Capital letters (A, B) refer to the mean number in a habitat regardless of month; lowercase letters (a, b) indicate differences between individual months.

**Table 1:** Number of individuals (IS) of beetles and their proportion (%) of the total number of carabid beetles in the FS and in adjacent fields, depending on the date of inventory (2024), in the species name, ‘*P*’ denotes predatory species, ‘*F*’ denotes phytophagous species, and the rest are hemizoophages.

		Location (Preferences to Habitats)											
No.	Species	Winter Wheat				Flower Strip				Rapeseed			
		May		June		May		June		May		June	
		IS	[%]	IS	[%]	IS	[%]	IS	[%]	IS	[%]	IS	[%]
1	*Agonum emarginatum ^P^*	0	0.00	0	0.00	1	0.50	0	0.00	0	0.00	0	0.00
2	*Amara aenea*	3	2.97	8	1.43	4	2.01	14	3.11	0	0.00	2	0.61
3	*Amara aulica*	0	0.00	0	0.00	1	0.50	1	0.22	0	0.00	0	0.00
4	*Amara bifrons*	0	0.00	1	0.18	1	0.50	4	0.89	0	0.00	0	0.00
5	*Amara curta*	0	0.00	0	0.00	0	0.00	0	0.00	0	0.00	0	0.00
6	*Amara familiaris*	0	0.00	0	0.00	3	1.51	1	0.22	0	0.00	0	0.00
7	*Amara littoralis*	0	0.00	0	0.00	0	0.00	0	0.00	0	0.00	0	0.00
8	*Amara ovata*	0	0.00	0	0.00	1	0.50	13	2.89	2	2.74	53	16.26
9	*Amara plebeja*	4	3.96	15	2.67	2	1.01	4	0.89	0	0.00	0	0.00
10	*Amara similata*	3	2.97	1	0.18	7	3.52	7	1.56	18	24.66	70	21.47
11	*Amara spreta*	0	0.00	0	0.00	0	0.00	0	0.00	0	0.00	0	0.00
12	*Amara tibialis*	0	0.00	0	0.00	1	0.50	0	0.00	0	0.00	0	0.00
13	*Amara tricuspidata*	1	0.99	342	60.96	1	0.50	7	1.56	0	0.00	0	0.00
14	*Anchomenus dorsalis ^P^*	0	0.00	5	0.89	3	1.51	8	1.78	0	0.00	3	0.92
15	*Anisodactylus binotatus*	0	0.00	0	0.00	0	0.00	1	0.22	0	0.00	0	0.00
16	*Anisodactylus signatus*	0	0.00	0	0.00	0	0.00	0	0.00	0	0.00	0	0.00
17	*Asaphidion flavipes ^P^*	0	0.00	0	0.00	0	0.00	0	0.00	0	0.00	0	0.00
18	*Bembidion lampros ^P^*	5	4.95	1	0.18	10	5.03	0	0.00	2	2.74	4	1.23
19	*Calathus ambiguus ^P^*	0	0.00	3	0.53	0	0.00	7	1.56	0	0.00	4	1.23
20	*Calathus erratus ^P^*	0	0.00	0	0.00	3	1.51	35	7.78	0	0.00	16	4.91
21	*Calathus fuscipes ^P^*	1	0.99	12	2.14	2	1.01	34	7.56	0	0.00	1	0.31
22	*Calathus melanocephalus ^P^*	0	0.00	1	0.18	6	3.02	3	0.67	0	0.00	0	0.00
23	*Demetrias atricapillus ^P^*	0	0.00	0	0.00	0	0.00	0	0.00	0	0.00	0	0.00
24	*Dolichus halensis ^P^*	0	0.00	0	0.00	0	0.00	1	0.22	0	0.00	0	0.00
25	*Dromius linearis ^P^*	0	0.00	0	0.00	1	0.50	0	0.00	0	0.00	0	0.00
26	*Harpalus affinis*	5	4.95	0	0.00	6	3.02	7	1.56	3	4.11	11	3.37
27	*Harpalus anxius*	2	1.98	0	0.00	1	0.50	0	0.00	0	0.00	0	0.00
28	*Harpalus distinguendus*	1	0.99	0	0.00	3	1.51	1	0.22	0	0.00	1	0.31
29	*Harpalus froelichii*	0	0.00	0	0.00	1	0.50	0	0.00	0	0.00	0	0.00
30	*Harpalus griseus*	0	0.00	0	0.00	0	0.00	2	0.44	0	0.00	0	0.00
31	*Harpalus pumilus*	0	0.00	0	0.00	0	0.00	0	0.00	0	0.00	0	0.00
32	*Harpalus rubripes*	0	0.00	2	0.36	2	1.01	0	0.00	0	0.00	0	0.00
33	*Harpalus rufipes*	2	1.98	5	0.89	22	11.06	19	4.22	2	2.74	30	9.20
34	*Harpalus signaticornis*	11	10.89	52	9.27	1	0.50	3	0.67	1	1.37	10	3.07
35	*Harpalus smaragdinus*	0	0.00	0	0.00	1	0.50	0	0.00	5	6.85	0	0.00
36	*Harpalus tardus*	0	0.00	0	0.00	1	0.50	1	0.22	0	0.00	2	0.61
37	*Harpalus xanthopus*	0	0.00	0	0.00	2	1.01	0	0.00	0	0.00	0	0.00
38	*Loricera pilicornis ^P^*	0	0.00	0	0.00	0	0.00	0	0.00	0	0.00	1	0.31
39	*Microlestes minutulus ^P^*	35	34.65	2	0.36	4	2.01	0	0.00	0	0.00	1	0.31
40	*Nebria brevicollis*	0	0.00	0	0.00	0	0.00	1	0.22	0	0.00	0	0.00
41	*Notiophilus germinyi ^P^*	0	0.00	0	0.00	0	0.00	0	0.00	0	0.00	0	0.00
42	*Ophonus laticollis*	0	0.00	0	0.00	0	0.00	0	0.00	0	0.00	1	0.31
43	*Poecilus cupreus ^P^*	25	24.75	80	14.26	92	46.23	216	48.00	40	54.79	98	30.06
44	*Poecilus lepidus ^P^*	1	0.99	10	1.78	0	0.00	0	0.00	0	0.00	2	0.61
45	*Poecilus punctulatus ^P^*	0	0.00	0	0.00	0	0.00	0	0.00	0	0.00	0	0.00
46	*Poecilus versicolor ^P^*	2	1.98	0	0.00	0	0.00	10	2.22	0	0.00	0	0.00
47	*Pterostichus melanarius ^P^*	0	0.00	19	3.39	16	8.04	50	11.11	0	0.00	16	4.91
48	*Pterostichus oblongopunctatus ^P^*	0	0.00	0	0.00	0	0.00	0	0.00	0	0.00	0	0.00
49	*Stomis pumicatus ^P^*	0	0.00	0	0.00	0	0.00	0	0.00	0	0.00	0	0.00
50	*Syntomus truncatellus ^P^*	0	0.00	0	0.00	0	0.00	0	0.00	0	0.00	0	0.00
51	*Trechus quadristriatus ^P^*	0	0.00	2	0.36	0	0.00	0	0.00	0	0.00	0	0.00
52	*Zabrus tenebrioides ^F^*	0	0.00	0	0.00	0	0.00	0	0.00	0	0.00	0	0.00
Individuals Species (Σ)		101		561		199		450		73		326	

**Table 2:** Number of individuals (IS) of beetles and their proportion (%) of the total number of carabid beetles in the FS and in adjacent fields of different crops to the strip, depending on the date of inventory (2025).

		Location (Preferences to Habitats)											
No.	Species	Wheat				Flower Strip				Rapeseed			
		May		June		May		June		May		June	
		IS	[%]	IS	[%]	IS	[%]	IS	[%]	IS	[%]	IS	[%]
1	*Agonum emarginatum*	0	0.00	0	0.00	0	0.00	0	0.00	0	0.00	0	0.00
2	*Amara aenea*	10	5.24	1	0.31	9	12.68	32	4.95	2	0.34	6	0.38
3	*Amara aulica*	0	0.00	0	0.00	0	0.00	10	1.55	1	0.17	0	0.00
4	*Amara bifrons*	0	0.00	2	0.62	1	1.41	14	2.16	1	0.17	1	0.06
5	*Amara curta*	0	0.00	0	0.00	0	0.00	3	0.46	0	0.00	1	0.06
6	*Amara familiaris*	0	0.00	0	0.00	0	0.00	1	0.15	0	0.00	0	0.00
7	*Amara littoralis*	0	0.00	0	0.00	0	0.00	0	0.00	0	0.00	2	0.13
8	*Amara ovata*	2	1.05	0	0.00	0	0.00	1	0.15	24	4.06	199	12.45
9	*Amara plebeja*	0	0.00	2	0.62	0	0.00	2	0.31	0	0.00	1	0.06
10	*Amara similata*	18	9.42	21	6.50	8	11.27	22	3.40	18	3.05	122	7.63
11	*Amara spreta*	0	0.00	0	0.00	0	0.00	1	0.15	0	0.00	0	0.00
12	*Amara tibialis*	0	0.00	1	0.31	0	0.00	0	0.00	0	0.00	0	0.00
13	*Amara tricuspidata*	1	0.52	1	0.31	2	2.82	117	18.08	0	0.00	2	0.13
14	*Anchomenus dorsalis*	7	3.66	45	13.93	2	2.82	24	3.71	22	3.72	97	6.07
15	*Anisodactylus binotatus*	0	0.00	0	0.00	0	0.00	1	0.15	0	0.00	0	0.00
16	*Anisodactylus signatus*	0	0.00	0	0.00	0	0.00	0	0.00	0	0.00	2	0.13
17	*Asaphidion flavipes*	0	0.00	1	0.31	0	0.00	0	0.00	0	0.00	0	0.00
18	*Bembidion lampros*	8	4.19	11	3.41	10	14.08	5	0.77	10	1.69	12	0.75
19	*Calathus ambiguus*	0	0.00	1	0.31	0	0.00	1	0.15	0	0.00	7	0.44
20	*Calathus erratus*	0	0.00	1	0.31	1	1.41	7	1.08	0	0.00	3	0.19
21	*Calathus fuscipes*	0	0.00	1	0.31	0	0.00	45	6.96	0	0.00	6	0.38
22	*Calathus melanocephalus*	0	0.00	0	0.00	0	0.00	3	0.46	0	0.00	0	0.00
23	*Demetrias atricapillus*	0	0.00	0	0.00	0	0.00	0	0.00	0	0.00	1	0.06
24	*Dolichus halensis*	0	0.00	7	2.17	0	0.00	5	0.77	0	0.00	3	0.19
25	*Dromius linearis*	0	0.00	0	0.00	0	0.00	0	0.00	0	0.00	0	0.00
26	*Harpalus affinis*	0	0.00	3	0.93	2	2.82	15	2.32	8	1.35	5	0.31
27	*Harpalus anxius*	0	0.00	0	0.00	0	0.00	1	0.15	0	0.00	1	0.06
28	*Harpalus distinguendus*	1	0.52	0	0.00	3	4.23	4	0.62	4	0.68	11	0.69
29	*Harpalus froelichii*	0	0.00	0	0.00	0	0.00	1	0.15	0	0.00	0	0.00
30	*Harpalus griseus*	0	0.00	0	0.00	0	0.00	0	0.00	0	0.00	0	0.00
31	*Harpalus pumilus*	0	0.00	0	0.00	0	0.00	0	0.00	1	0.17	0	0.00
32	*Harpalus rubripes*	0	0.00	0	0.00	0	0.00	1	0.15	0	0.00	1	0.06
33	*Harpalus rufipes*	2	1.05	23	7.12	15	21.13	82	12.67	6	1.02	46	2.88
34	*Harpalus signaticornis*	1	0.52	6	1.86	0	0.00	6	0.93	11	1.86	71	4.44
35	*Harpalus smaragdinus*	0	0.00	0	0.00	1	1.41	0	0.00	0	0.00	21	1.31
36	*Harpalus tardus*	1	0.52	0	0.00	1	1.41	4	0.62	0	0.00	0	0.00
37	*Harpalus xanthopus*	0	0.00	0	0.00	0	0.00	0	0.00	0	0.00	0	0.00
38	*Loricera pilicornis*	0	0.00	0	0.00	0	0.00	0	0.00	1	0.17	1	0.06
39	*Microlestes minutulus*	7	3.66	0	0.00	3	4.23	5	0.77	0	0.00	0	0.00
40	*Nebria brevicollis*	0	0.00	0	0.00	0	0.00	0	0.00	0	0.00	0	0.00
41	*Notiophilus germinyi*	0	0.00	1	0.31	0	0.00	0	0.00	0	0.00	0	0.00
42	*Ophonus laticollis*	0	0.00	0	0.00	0	0.00	0	0.00	0	0.00	0	0.00
43	*Poecilus cupreus*	122	63.87	170	52.63	8	11.27	147	22.72	474	80.20	960	60.08
44	*Poecilus lepidus*	9	4.71	7	2.17	1	1.41	9	1.39	6	1.02	1	0.06
45	*Poecilus punctulatus*	0	0.00	0	0.00	0	0.00	0	0.00	1	0.17	0	0.00
46	*Poecilus versicolor*	0	0.00	0	0.00	3	4.23	15	2.32	1	0.17	2	0.13
47	*Pterostichus melanarius*	2	1.05	17	5.26	0	0.00	58	8.96	0	0.00	13	0.81
48	*Pterostichus oblongopunctatus*	0	0.00	0	0.00	0	0.00	1	0.15	0	0.00	0	0.00
49	*Stomis pumicatus*	0	0.00	0	0.00	0	0.00	1	0.15	0	0.00	0	0.00
50	*Syntomus truncatellus*	0	0.00	0	0.00	1	1.41	1	0.15	0	0.00	0	0.00
51	*Trechus quadristriatus*	0	0.00	1	0.31	0	0.00	0	0.00	0	0.00	0	0.00
52	*Zabrus tenebrioides*	0	0.00	0	0.00	0	0.00	2	0.31	0	0.00	0	0.00
Individuals Species (Σ)		191		323		71		647		591		1598	

**Table 3:** The average number of individuals in a trap per habitat depending on year.

Habitat	$\bar{x}$ (SD)	Me (Min./Max.)	Mean Rank	Kruskal–Wallis Test
				(2, *N* = 60)
2024				
Winter wheat field	16.55 (±35.17)	1.25 (0.00/133.50)	27.15	H = 6.724
Flower strip	16.23 (±16.40)	10.50 (0.00/63.50)	38.55	*p* = 0.0347
Rapeseed field	9.98 (±16.12)	0.50 (0.00/72.00)	25.80	
Test statistics	χ^2^ = 8.94 df = 2 *p* = 0.0114			
Significant difference not significant				
2025				
Winter wheat field	12.85 (±18.76)	0.50 (0.00/81.00)	24.88	H = 3.586
Flower strip	17.95 (±23.43)	6.00 (0.50/87.00)	34.93	*p* = 0.1665
Rapeseed field	54.73 (±73.93)	6.50 (0.00/306.00)	31.70	
Test statistics	χ^2^ = 1.60 df = 2 *p* = 0.4493			
Significant difference not significant				

Me—mediana. The mean rank is the average rank of observations in a given group.

**Table 4:** The number of individuals in the family of Carabidae beetles, by habitats, survey date and per one trap.

Habitat	Month	
	May	June
2024		
Winter wheat field	5.05 (±6.96) b	28.05 (±43.03) a
Flower strip	9.95 (±8.32) a	22.50 (±17.73) a
Rapeseed field	3.65 (±8.26) b	16.30 (±22.42) a
mean	6.22 (±8.20) B	22.28 (±29.71) A
2025		
Winter wheat field	9.55 (±11.83) a	16.15 (±28.52) a
Flower strip	3.55 (±1.95) b	32.35 (±26.59) a
Rapeseed field	29.55 (±42.35) a	79.90 (±98.67) a
mean	14.22 (±27.38) A	42.80 (±66.10) A

Average marked with the same letters do not differ significantly at α = 0.05. Capital letters (A, B) refer to the mean number between individual months, regardless of type of habitat, lowercase letters (a, b) indicate differences between habitats.

## Data Availability

The data presented in this study are available on request from the corresponding authors.
